# Patient‐reported outcome thresholds and their associations with survival, adverse events, and quality of life in a pooled analysis of breast cancer trials

**DOI:** 10.1002/ijc.70020

**Published:** 2025-06-21

**Authors:** Bradley D. Menz, Natansh D. Modi, Ahmad Y. Abuhelwa, Nicole M. Kuderer, Gary H. Lyman, Sandra M. Swain, Ganessan Kichenadasse, Adel Shahnam, Mark Haseloff, Agnes Vitry, Elke Rammant, Imogen Ramsey, Raymond J. Chan, Ross A. McKinnon, Andrew Rowland, Michael J. Sorich, Ashley M. Hopkins

**Affiliations:** ^1^ College of Medicine and Public Health, Flinders Health and Medical Research Institute Flinders University Adelaide Australia; ^2^ Department of Pharmacy Practice and Pharmacotherapeutics, College of Pharmacy University of Sharjah Sharjah United Arab Emirates; ^3^ Advanced Cancer Research Group Kirkland Washington USA; ^4^ Department of Public Health Sciences Fred Hutchinson Cancer Center Seattle Washington USA; ^5^ Department of Medicine Duke University School of Medicine Durham North Carolina USA; ^6^ Georgetown Lombardi Comprehensive Cancer Center and MedStar Health Washington DC USA; ^7^ Flinders Centre for Innovation in Cancer, Department of Medical Oncology, Flinders Medical Centre Flinders University Bedford Park South Australia Australia; ^8^ Medical Oncology Peter MacCallum Cancer Centre Melbourne Australia; ^9^ Consumer Advisory Group, Clinical Cancer Epidemiology Group, College of Medicine and Public Health, Flinders Health and Medical Research Institute Flinders University Adelaide Australia; ^10^ University of South Australia Clinical and Health Sciences Adelaide Australia; ^11^ Department of Human Structure and Repair Ghent University Ghent Belgium; ^12^ Caring Futures Institute, College of Nursing and Health Sciences Flinders University Adelaide Australia

**Keywords:** breast cancer, cancer, EORTC, patient‐reported outcomes, survival outcomes

## Abstract

Researchers at the EORTC recently recommended clinical thresholds for the QLQ‐C30 to facilitate actionable insights in clinical practice. We evaluate the distribution of these thresholds and associations with outcomes in breast cancer. Data were pooled from two early‐stage and six advanced‐stage breast cancer trials. EORTC thresholds were applied to available QLQ‐C30 data to identify clinically important PRO domains. Associations between the number of clinically important PRO domains at baseline with overall survival (OS), invasive‐disease‐free survival (IDFS), progression‐free survival (PFS), grade ≥3 adverse events (AEs), and serious AEs were evaluated using Cox‐regression. Data from 8544 breast cancer patients, of whom 2428 (41%) of the 5893 early‐stage and 1486 (56%) of the 2651 advanced‐stage patients reported ≥3 clinically important PRO domains. In the early‐stage, each additional clinically important PRO domain was associated with worsened grade ≥3 AEs (HR, 1.03 [95%CI, 1.01–1.04], *p* = 0.001) and serious AEs (1.05 [1.03–1.07], *p* < 0.001). In the advanced‐stage, each additional clinically important PRO domain was associated with worsened OS (1.05 [1.03–1.07], *p* < 0.001), PFS (1.03 [1.01–1.04], *p* = 0.002), grade ≥3 AEs (1.04 [1.02–1.06], *p* < 0.001), and serious AEs (1.07 [1.04–1.11], *p* < 0.001). A substantial proportion of breast cancer patients report clinically important PRO domains at baseline, with increasing numbers associated with worsening AEs, survival, and quality‐of‐life.

## What's New?

The Quality of Life Questionnaire Core 30 (QLQ‐C30) is useful for assessing patient perspectives on the impact of cancer treatments on quality of life. However, thresholds to determine when patients are experiencing functional or symptomatic burdens remain under explored. In this meta‐analysis, the authors evaluated relationships between QLQ‐C30 thresholds and clinical outcomes in breast cancer. Analyses show that more than 40 percent of patients reported impacts in three or more clinically important domains, including physical function, emotional function, and financial difficulty. The more clinically important domains reported, the greater the likelihood of adverse events, worse survival, and reduced quality‐of‐life.

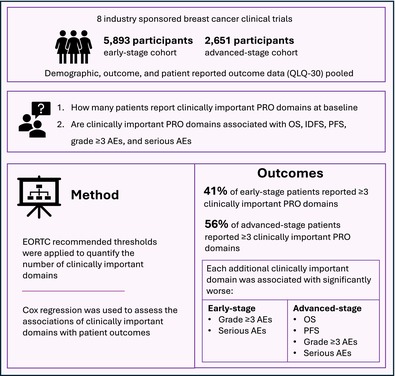

**Abbreviations**
AE: Adverse Event
ECOG‐PS: Eastern Cooperative Oncology Group Performance Status
EORTC: European Organisation for Research and Treatment of Cancer
ER: Estrogen Receptor
HER2: Human Epidermal Growth Factor Receptor 2
HR: Hazard Ratio
IDFS: Invasive Disease‐Free Survival
IQR: Interquartile Range
MSE: Mean Squared Error
OS: Overall Survival
PFS: Progression‐Free Survival
PR: Progesterone Receptor
PRO: Patient‐Reported Outcome
QLQ‐C30: Quality of Life Questionnaire‐Core 30
QoL: Quality of Life
SHAP: SHapley Additive exPlanations

## 1 INTRODUCTION

Patient‐reported outcomes (PROs) are health outcomes reported directly by patients, through validated questionnaires. PROs can capture the impacts of a health condition or its treatment on health‐related quality of life (QoL), as well as symptoms and physical, social, and emotional functioning.^1^, ^2^, ^3^ PROs are increasingly recognised for their potential to enhance shared decision‐making between patients and clinicians.^4^, ^5^, ^6^, ^7^ This includes randomised controlled trials that have demonstrated that electronic symptom monitoring using PROs can help identify actionable PRO insights that lead to improved QoL, reduced toxicity, and improved survival outcomes in patients with advanced cancers.^8^, ^9^, ^10^ However, despite their potential, PROs are not yet widely implemented outside of clinical trials. Several barriers limit their integration into routine practice, including a lack of large‐scale data, particularly for individual cancer types, and challenges in determining which specific PRO questionnaires, among the many available, are most clinically useful.^11^, ^12^, ^13^, ^14^ Additional considerations include the resources required for administering PROs and the limited time clinicians have in busy practice settings.^15^

The European Organisation for Research and Treatment of Cancer (EORTC) Quality of Life Questionnaire Core 30 (QLQ‐C30) is the most widely used PRO instrument in cancer trials to assess the impact of treatments on patients' QoL.^16^, ^17^, ^18^ Considerable efforts have been made to facilitate the clinical application of the EORTC QLQ‐C30,^19^, ^20^ including studies that have analysed the distribution of QLQ‐C30 responses across various cancer types and disease stages.^17^, ^21^, ^22^, ^23^ Recently, researchers at the EORTC recommended clinically important thresholds for the QLQ‐C30 to help clinicians more easily identify actionable insights from PROs, by making it clearer when patients are experiencing functional or symptomatic burdens.^24^, ^25^ However, this study was limited by a small sample size and the inclusion of multiple cancer types, which reduces its applicability to patients with breast cancer.^24^ Therefore, it is important to evaluate these thresholds in larger, more focused cohorts, such as patients with breast cancer. Additionally, understanding the relationship between clinically important PRO domains and key cancer outcomes—such as survival, disease progression, toxicity, and QoL—would provide valuable insights.

The primary aim of this study was to determine the frequency of patients reporting clinically important PRO domains before treatment initiation (i.e., pre‐treatment/baseline) in early‐stage and advanced‐stage breast cancer. The secondary aim was to assess the association between the cumulative number of clinically important domains with overall survival (OS), invasive‐disease‐free survival (IDFS), progression‐free survival (PFS), grade ≥3 adverse events (AEs), serious AEs, and to explore which of the QLQ‐C30 domains were most associated with QoL at baseline.

## 2 METHODS

This publication is based on research using data from Lilly, Pfizer, and Roche that has been made available through Vivli, inc.^26^ Vivli has not contributed to or approved, and is not in any way responsible for, the contents of this publication. Anonymised clinical trial individual participant data were accessed through Vivli to evaluate predictors, including PROs, for their ability to identify the likelihood of adverse events and therapeutic outcomes in patients with breast cancer. The team received access to analysis‐ready and raw datasets, via Vivli, that had redactions and anonymizations to protect patient confidentiality. This study includes all clinical trials available to our team that assessed QoL using the EORTC QLQ‐C30.

### 2.1 Clinical trials

The study included two early‐stage breast cancer clinical trials, APHINITY (NCT01358877, data cut: December 2016)^27^ and KATHERINE (NCT01772472, data cut: July 2018).^28^ Both trials had participants with human epidermal growth factor receptor 2 (HER2)‐positive, operable, non‐metastatic, adequately excised, primary invasive breast cancer. Further, six advanced‐stage trials were included: MONARCH 1 (NCT02102490, data cut: October 2016),^29^ MONARCH 2 (NCT02107703, data cut: February 2017),^30^ MONARCH 3 (NCT02246621, data cut: November 2017),^31^ NEXTMONARCH (NCT02747004, data cut: June 2020),^32^ PALOMA3 (phase III trial, NCT01942135, data cut: October 2015)^33^ and TH3RESA (NCT01419197, data cut: August 2015).^34^ The MONARCH trials and PALOMA3 included participants with hormone receptor‐positive, HER2‐negative breast cancer who had not received prior systemic therapy in the advanced setting and had metastatic disease or locoregional recurrence not suitable for surgery or radiotherapy. TH3RESA included participants with HER2‐positive breast cancer who had received prior treatment and had unresectable, locally advanced, recurrent, or metastatic breast cancer.

### 2.2 Patient‐reported outcome data

Across all evaluated clinical trials, PROs were assessed using the EORTC QLQ‐C30, version 3.0. The QLQ‐C30 is a validated instrument comprising of 30 items that measure functional outcomes and symptoms across 15 distinct domains.^35^, ^36^ These include five functional domains (physical, role, emotional, cognitive, and social functioning), nine symptom domains (fatigue, pain, nausea and vomiting, dyspnoea, appetite loss, insomnia, constipation, diarrhea, and financial difficulties), and a global health status (i.e., QoL) domain.^35^, ^36^ The 28 items assessing functional and symptom questions are scored using a 4‐point Likert scale, with responses ranging from 1 (“Not at all”) to 4 (“Very much”). The two items assessing global health status (i.e., QoL) are scored on a 7‐point Likert scale, with responses ranging from 1 (“Very poor”) to 7 (“Excellent”). In the clinical trial data utilised for this study, these raw responses were converted into standardised domain scores ranging from 0 to 100, according to the EORTC scoring manual.^35^ For the functional and QoL domains, lower scores indicate poorer function or QoL, whereas for symptom domains, higher scores signify greater symptom burden.^16^ To classify the domains as “clinically important”, thresholds of clinical importance recommended by the EORTC were applied. A domain was deemed “clinically important” if the score indicated significant functional impairment or symptom burden.^24^, ^25^ Specifically, a functional domain was classified as clinically important when its score fell below the threshold, while a symptom domain was classified as clinically important when its score exceeded the threshold.^24^, ^25^ Table S1 details the thresholds used in the study to define clinically important PRO domains.

### 2.3 Outcomes data

Across all evaluated trials, OS was defined as the time from randomisation/first dose to death from any cause. For the early‐stage trials, IDFS was defined as the time from randomisation to the date of first occurrence of: ipsilateral invasive breast tumor recurrence; ipsilateral local‐regional invasive breast cancer recurrence; distant recurrence; death attributable to any cause; and contralateral invasive breast cancer.^27^, ^28^ For the advanced‐stage trials, PFS was defined as the time from randomisation/first dose to progression, according to Response Evaluation Criteria in Solid Tumors (RECIST) v 1.1, or death from any cause.^29^, ^30^, ^31^, ^32^, ^33^, ^34^ Event censorings occurred at the last known follow‐up date. Grade ≥3 AEs of any cause were classified in accordance with National Cancer Institute Common Terminology Criteria for Adverse Events (NCI‐CTCAE) version 4.0.^27^, ^28^, ^29^, ^30^, ^31^, ^32^, ^33^, ^34^ Serious AEs were classified when AEs: resulted in death, were life‐threatening, required inpatient hospitalization or prolonged hospitalization, caused persistent or significant disability/incapacity, or resulted in a congenital anomaly/birth defect.^37^

### 2.4 Statistical analysis

The frequency of patients reporting clinically important PRO domains at baseline was presented using descriptive statistics for both the early and advanced disease cohorts. Chi‐square tests were used to evaluate frequency differences between the early and advanced disease cohorts based on a two‐tailed *p*‐value threshold of <0.05 for significance.

Using a pooled one‐stage individual‐participant data approach,^38^ Cox proportional hazards analysis was used to evaluate the relationship between the number of clinically important PRO domains at baseline with outcomes including OS, IDFS, PFS, grade ≥3 AEs, and serious AEs. Associations were presented as hazard ratios (HRs) with 95% confidence intervals (95% CIs). All analyses were adjusted for potential confounders, including age, weight, Eastern Cooperative Oncology Group Performance Status (ECOG‐PS), HER2 status, estrogen receptor status, progesterone receptor status, comorbidity count, line of therapy, and by study. Non‐linear associations were assessed using cut points, cubic splines, and the Akaike Information Criterion (AIC). Kaplan–Meier curves were used to plot the identified associations between the number of clinically important domains and each of the evaluated outcomes. Missing data were handled using complete case analyses, wherein participants with complete PRO, adjustment variable, and outcome data were included in the analysis. As a sensitivity analysis, two‐stage meta‐analyses were conducted to evaluate the consistency of identified associations with the one‐stage approach and across the pooled clinical trials.^38^ Additional sensitivity analyses were conducted to assess the associations between each individual clinically important PRO domain and OS, IDFS, PFS, and AEs.

To explore how each baseline functional and symptom domain of the QLQ‐C30 influenced individuals' ratings of their QoL domain (i.e., global health status), a random forest prediction model was developed. The random forest approach was chosen for its ability to manage complex, non‐linear interactions between variables. The random forest prediction model was developed using the R package “Ranger”, with default settings: 500 trees, mtry equal to the square root of the number of variables randomly sampled at each split, and a minimum node size of 5. Model performance was assessed by the *R* ^2^, mean square error (MSE), and visualised using a calibration curve comparing the mean predicted QoL scores generated by the model with the average QoL scores reported by patients. Feature importance was determined using permutation, and out‐of‐bag sampling was employed to avoid model overfitting.^39^ From this model, Shapley Additive Explanation (SHAP) values were calculated to create a SHAP bee swarm plot. This plot ranks the importance of each feature (e.g., PRO domain) and its impact on QoL.

All statistical analyses and machine learning modelling were conducted using R version 4.3.3.

## 3 RESULTS

### 3.1 Patient population

In this pooled analysis of eight clinical trials, a total of 8544 patients were included, with 5893 in the early‐stage cohort and 2651 in the advanced‐stage cohort. The median follow‐up time was 49 months in the early‐stage cohort and 20 months in the advanced‐stage cohort. Table S2 provides a summary of the baseline characteristics for both cohorts, where it was observed that no characteristics had more than 4% missing data.

Patients in the advanced‐stage cohort were older, more likely to have ECOG‐PS scores of 1 or higher, had a higher incidence of hormone receptor‐positive disease, and had more comorbidities compared to those in the early‐stage cohort (Table S2). Detailed information on treatment regimens and demographic characteristics for both cohorts is available in Tables S3 and S4. Briefly, in the early‐stage cohort, 5162 patients were randomised to receive trastuzumab (with chemotherapy or alone) ± pertuzumab and 731 were randomised to receive trastuzumab emtansine. In the advanced‐stage cohort, patients were randomised to receive one of the following: fulvestrant or non‐steroidal aromatase inhibitor or tamoxifen with or without abemaciclib (*n* = 1140); fulvestrant with or without palbociclib (*n* = 744); trastuzumab emtansine (*n* = 404); physicians' choice (*n* = 198); or a non‐steroidal aromatase inhibitor alone (*n* = 165).

Table S5 presents the median values (interquartile range, IQR) for each PRO domain in the evaluated early‐ and advanced‐stage cohorts, which are similar to the EORTC QLQ‐C30 reference values for these domains in the respective cohorts.^40^ Across both the early and advanced‐stage cohorts, no PRO domains had more than 10% missing data pre‐treatment (Table S5).

### 3.2 Clinically important pre‐treatment PRO domains

As defined by the EORTC‐recommended thresholds, Table 1 presents the frequency of clinically important pre‐treatment PROs experienced for each individual PRO domain in the early‐ and advanced‐stage cohorts. Table 2 summarizes the characteristics of the aggregate number of clinically important pre‐treatment PRO domains across the early‐ and advanced‐stage cohorts. In the early‐stage cohort, 4362 (74%) reported one or more clinically important PRO domains, and 2428 (41%) reported three or more. The most frequently observed clinically important domains in this cohort were emotional function (*n* = 2494; 42%), financial difficulties (*n* = 2339; 40%), pain (*n* = 2036; 35%), physical function (*n* = 1393; 24%), and cognitive function (*n* = 1133; 19%). In the advanced‐stage cohort, 2118 (80%) patients reported one or more clinically important PRO domains, and 1486 (56%) patients reported three or more. The most frequently observed clinically important PRO domains in this cohort were physical function (*n* = 1349; 51%), pain (*n* = 1241; 47%), emotional function (*n* = 1052; 40%), financial difficulties (*n* = 1007; 38%), and fatigue (*n* = 852; 32%). Tables S6 and S7 presents a summary of the demographics for patients reporting one or more clinically important PRO domains prior to treatment initiation within their respective clinical trial.

**TABLE 1:** Baseline frequency of clinically important PRO domains in the early‐ and advanced‐stage cohorts.

	Early‐stage cohort (*n* = 5893)	Advanced‐stage cohort (*n* = 2651)	*p*‐value
*Number (%) of patients who have clinically important patient reported outcome domains*			
Physical function			<0.001
Clinically important	1393 (24%)	1349 (51%)	
Not clinically important	4162 (71%)	1094 (41%)	
Missing	338 (6%)	208 (8%)	
Role function			<0.001
Clinically important	900 (15%)	576 (22%)	
Not clinically important	4646 (79%)	1866 (70%)	
Missing	347 (6%)	209 (8%)	
Emotional function			0.13
Clinically important	2494 (42%)	1052 (40%)	
Not clinically important	3053 (52%)	1389 (52%)	
Missing	346 (6%)	210 (8%)	
Cognitive function			< 0.001
Clinically important	1133 (19%)	665 (25%)	
Not clinically important	4417 (75%)	1775 (67%)	
Missing	343 (6%)	211 (8%)	
Social function			<0.001
Clinically important	789 (13%)	476 (18%)	
Not clinically important	4754 (81%)	1962 (74%)	
Missing	350 (6%)	213 (8%)	
Fatigue			<0.001
Clinically important	956 (16%)	852 (32%)	
Not clinically important	4595 (78%)	1588 (60%)	
Missing	342 (6%)	211 (8%)	
Nausea and vomiting			<0.001
Clinically important	748 (13%)	641 (24%)	
Not clinically important	4807 (82%)	1802 (68%)	
Missing	338 (6%)	208 (8%)	
Pain			<0.001
Clinically important	2036 (35%)	1241 (47%)	
Not clinically important	3518 (60%)	1202 (45%)	
Missing	339 (6%)	208 (8%)	
Dyspnoea			<0.001
Clinically important	1192 (20%)	1057 (40%)	
Not clinically important	4350 (74%)	1381 (52%)	
Missing	351 (6%)	213 (8%)	
Insomnia			<0.001
Clinically important	1068 (18%)	560 (21%)	
Not clinically important	4478 (76%)	1883 (71%)	
Missing	347 (6%)	208 (8%)	
Appetite loss			<0.001
Clinically important	217 (4%)	306 (12%)	
Not clinically important	5333 (90%)	2136 (81%)	
Missing	343 (6%)	209 (8%)	
Constipation			<0.001
Clinically important	257 (4%)	241 (9%)	
Not clinically important	5292 (90%)	2198 (83%)	
Missing	344 (6%)	212 (8%)	
Diarrhoea			<0.001
Clinically important	834 (14%)	456 (17%)	
Not clinically important	4709 (80%)	1982 (75%)	
Missing	350 (6%)	213 (8%)	
Financial toxicity			0.34
Clinically important	2339 (40%)	1007 (38%)	
Not clinically important	3159 (54%)	1428 (54%)	
Missing	395 (7%)	216 (8%)	

**TABLE 2:** Aggregate number of clinically important patient‐reported outcome domains across the early and advanced‐stage cohorts.

	Early‐stage cohort (*n* = 5893)	Advanced‐stage cohort (*n* = 2651)	*p*‐value
Median number of clinically important patient‐reported outcome domains by participants			
Median (range; IQR)	2 (0–14; 1–4)	4 (0–14; 1–7)	
Missing	438 (7%)	233 (9%)	
Number of clinically important patient‐reported outcome domains by participants			<0.001
0	1093 (19%)	300 (11%)	
1	1103 (19%)	349 (13%)	
2	831 (14%)	283 (11%)	
3+	2428 (41%)	1486 (56%)	
Missing	438 (7%)	233 (9%)	

Abbreviation: IQR, interquartile range.

### 3.3 Associations of clinically important PRO domains with survival, progression, and adverse events

Table 3 presents the results of the one‐stage Cox proportional hazard analysis evaluating the association between the number of clinically important PRO domains with OS, IDFS/PFS, grade ≥3 AEs, and serious AEs in both the early‐stage and advanced‐stage cohorts. The relationships observed were best described as linear across all analyses.

**TABLE 3:** Associations between the number of clinically important patient‐reported outcome domains and overall survival, progression, and adverse events by disease stage.

Event outcome	Early‐stage cohort^a^			Advanced‐stage cohort^a^		
	*n*/*N*	HR (95% CI)^b^	*p*	*n*/*N*	HR (95% CI)^b^	*p*
Overall survival	216/5377	0.98 (0.93 to 1.03)	0.32	852/2301	1.05 (1.03 to 1.07)	<0.001
Invasive‐disease‐free/progression‐free survival^c^	516/5377	1 (0.97 to 1.03)	0.99	1355/2301	1.03 (1.01 to 1.04)	0.0019
Grade ≥3 adverse events	2752/5377	1.03 (1.01 to 1.04)	0.001	1124/2301	1.04 (1.02 to 1.06)	<0.001
Serious adverse events	1241/5377	1.05 (1.03 to 1.07)	<0.001	385/2301	1.07 (1.04 to 1.11)	<0.001

Abbreviations: HR, hazard ratio; 95% CI, 95% confidence interval; n/N, number of events/number of cohort population.

^a^ Analyses adjusted for age, body mass index, race, Eastern Cooperative Oncology Group—performance status, Human epidermal growth factor receptor 2 status, Estrogen receptor status, Progesterone receptor status, comorbidity count, and line of therapy.

^b^ Hazard ratio represents change per unit increase of clinically important PRO domains.

^c^ Invasive‐disease‐free survival assessed in the early stage cohort and progression free survival assessed in the advanced stage cohort.

**FIGURE 1:**
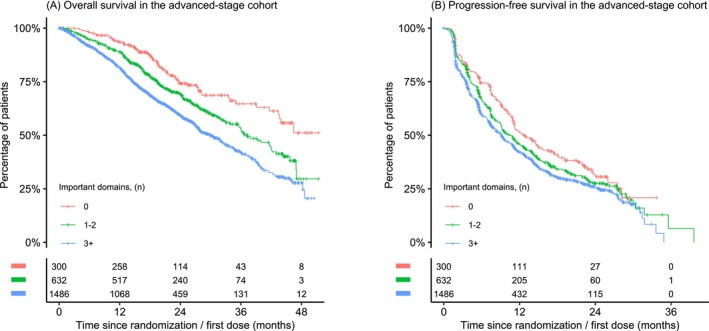
Kaplan–Meier estimates of (A) overall survival and (B) progression‐free survival outcomes in the advanced‐stage cohort by the number of clinically important patient‐reported outcome domains.

**FIGURE 2:**
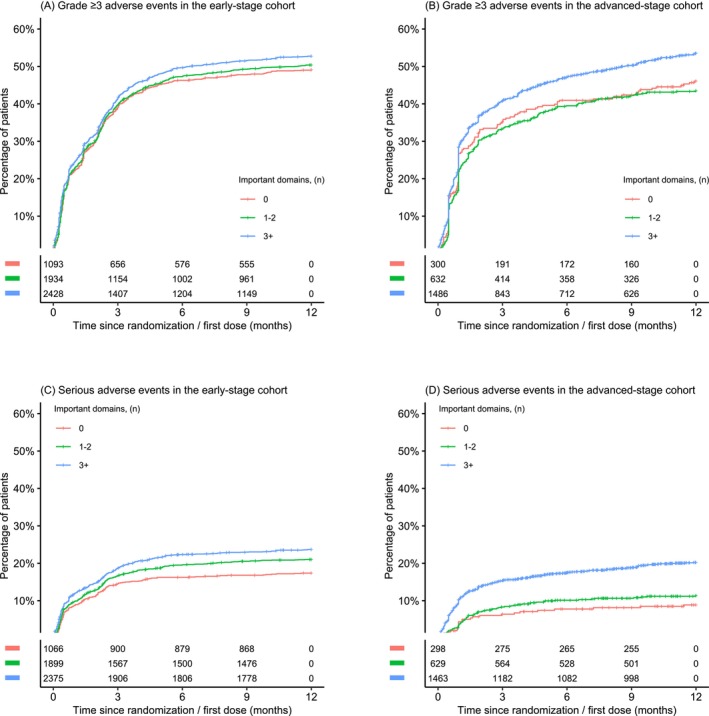
Kaplan–Meier estimates of (A) grade ≥3 adverse events and (C) serious adverse events by the number of clinically important patient‐reported outcome domains in the early‐stage cohort. Kaplan–Meier estimates of (B) grade ≥3 adverse events and (D) serious adverse events by the number of clinically important patient‐reported outcome domains in the advanced‐stage cohort.

In the early‐stage cohort, each additional clinically important PRO domain was significantly associated with an increased risk of grade ≥3 AEs (HR, 1.03 [95%CI, 1.01 to 1.04], *p* < 0.001) and serious AEs (HR, 1.05 [95%CI, 1.03 to 1.07], *p* < 0.001). In the early‐stage cohort, no significant associations were found between additional clinically important PRO domains and IDFS or OS. In the advanced‐stage cohort, each additional clinically important PRO domain was significantly associated with decreased OS (HR, 1.05 [95% CI 1.03 to 1.07], *p* < 0.001), decreased PFS (HR, 1.03 [95% CI 1.01 to 1.04], *p* < 0.002), increased grade ≥3 AEs (HR, 1.04 [95% CI 1.02 to 1.06], *p* < 0.001), and increased serious AEs (HR, 1.07 [95% CI 1.04 to 1.11], *p* < 0.001). The relationships between the cumulative number of clinically important PRO domains and assessed outcomes are depicted in Figures 1, 2, and S1 using Kaplan–Meier plots. Absolute differences in outcomes are detailed in Table S8. No substantial differences in the identified associations were observed with a two‐stage meta‐analysis approach, nor between the clinical trials via the two‐stage approach (Figures S2–S9). Associations between each of the individual clinically important PRO domains and OS, IDFS, PFS, and AEs are provided in Tables S9–S12.

### 3.4 Machine learning prediction of QoL

**FIGURE 3:**
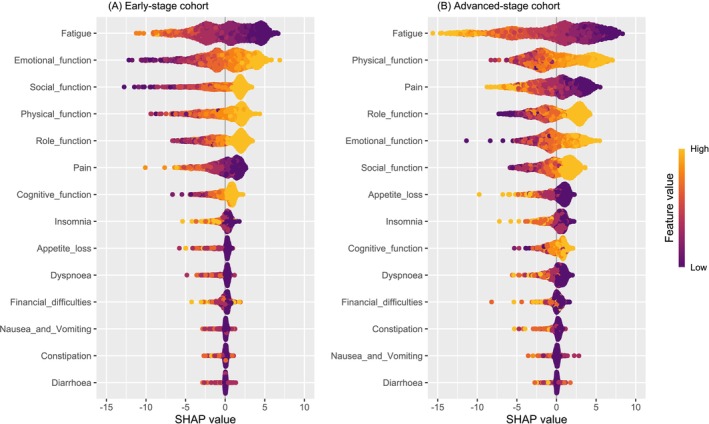
Shapley Additive Explanations (SHAP) bee swarm plot of the random forest regression model by (A) the early‐stage cohort and (B) the advanced‐stage cohort. Each point represents a SHAP value for an individual patient. The x‐axis position of each point (SHAP value) represents the influence of the feature (i.e., PRO domain) on the model output (quality of life).

A random forest prediction model was developed ranking the influence of each baseline PRO domain on baseline patient‐reported QoL for both the early‐stage (*R* ^2^: 0.43; MSE: 217) and advanced‐stage (*R* ^2^: 0.49; MSE: 264) cohorts. Calibration curves for the model are provided in Figure S10. SHAP analysis of the model indicated that the most influential PRO domains for predicting baseline QoL in the early‐stage cohort were fatigue, emotional function, social function, physical function, and role function. In the advanced‐stage cohort, the most influential domains were fatigue, physical function, pain, role function and emotional function. Figure 3. presents the importance scores of the PRO domains for predicting QoL, and the directional impact each domain has on patient reported QoL.

## 4 DISCUSSION

To the best of the authors' knowledge, this study represents the largest analysis to date of EORTC QLQ‐C30 data in breast cancer. It is the first study to quantify the prevalence of clinically important PRO domains in patients with early and advanced breast cancer according to validated clinical thresholds for EORTC QLQ‐C30 scales, and the first to demonstrate the associations of these clinically important PRO domains with survival and AE outcomes. In the early‐stage cohort, 41% of patients reported three or more clinically important PRO domains, and it was shown that an increasing number of clinically important PRO domains was significantly associated with higher AE risks. In the advanced‐stage cohort, 56% of patients reported three or more clinically important PRO domains, with each additional clinically important PRO domain being associated with worsening OS, PFS, and AE risks. Across the pooled population, emotional function (42%), financial difficulty (39%), pain (38%), physical function (32%), and dyspnoea (26%) were the PRO domains most frequently reported as clinically important in patients with breast cancer, while machine learning demonstrated that these PRO domains were linked to influences on overall QoL.

Randomised controlled trials have demonstrated that the routine collection and monitoring of PROs in individuals with advanced cancer can enable timely clinician interventions, leading to improved QoL, OS, reduced hospital visits, and fewer AEs.^8^, ^9^, ^10^ Although these findings are promising, the trials included a heterogeneous mix of cancer types, with <20% of participants having breast cancer, and none with early‐stage disease.^8^, ^9^, ^10^ Consequently, while these trials highlight substantial potential benefits from routine PRO collection, PROs are not yet widely used in routine practice. Further evidence is needed to understand the potential of PROs in breast cancer, particularly in the early‐stage setting. In this study, we found that over 40% of breast cancer patients with both early‐ and advanced‐stage disease reported three or more clinically important PRO domains at baseline, with the most frequently clinically important domains being emotional function, financial difficulty, pain, physical function, and dyspnoea. Overall, these findings emphasise the potential of applying clinical thresholds to EORTC QLQ‐C30 data for identifying clinically actionable PRO insights.

Prior research using observational data has demonstrated that PROs are consistently associated with survival, AE, and QoL outcomes in patients with breast, lung, ovarian, gastrointestinal, and urothelial cancers.^41^, ^42^, ^43^, ^44^, ^45^, ^46^, ^47^, ^48^ This study confirms that in patients with advanced‐stage breast cancer, an increasing number of clinically important PRO domains is associated with worsening survival and AE risks. In early‐stage disease, AE risks also worsened as the number of clinically important PRO domains increased. Furthermore, with machine learning, it was found that the most influential PRO domain affecting overall QoL was fatigue, consistent with prior reports in the literature that fatigue is a major driver of QoL in patients with breast cancer.^49^, ^50^ Additionally, it was observed that for the early‐stage disease cohort, the five domains of the QLQ‐C30 most associated with QoL were fatigue, emotional function, social function, physical function, and role function. While for the QoL of patients with advanced disease, the key PRO domains were fatigue, physical function, pain, role function, and emotional function. Overall, these findings suggest that prospective clinical trials are warranted to determine whether interventions addressing clinically important PRO domains can subsequently improve QoL, survival, and AE outcomes for breast cancer patients.

This study utilised IPD from eight industry‐sponsored clinical trials, providing access to large, high‐quality, rigorously collected PRO, AE, and survival data.^51^, ^52^, ^53^ Although the pooled dataset contained minimal missing PRO data, we acknowledge that the use of complete‐case analysis may introduce biases. Similarly, there are limitations regarding the generalizability of our findings to real‐world populations. For example, the strict inclusion criteria used in these trials—such as the limited recruitment of patients with poor performance status and that over 90% of participants were of White or Asian race—may limit the applicability of our findings to more diverse populations. Additionally, the absence of lymph node status, tumour size, locoregional treatments, menopause status, and grade data across each of the pooled trials limited our ability to adjust for these factors. For the early‐stage cohort, it should also be appreciated that all participants had HER2‐positive disease; with the APINITY trial assessing anticancer treatment in the adjuvant setting, while KATHERINE focused on adjuvant treatment for participants with residual invasive disease in the breast or axilla at surgery after receiving neoadjuvant therapy. Moreover, the timing between locoregional treatments and baseline PRO collection may have varied, potentially influencing PRO measures between participants. Lastly, it should be noted that no participants in this pooled study had triple‐negative breast cancer, and that 77% of the advanced‐stage cohort were ER/PR‐positive, HER2‐negative.

The developed QoL prediction model demonstrated modest explanatory power (as indicated by the *R* ^2^) and prediction accuracy (as indicated by the MSE). Further, the identified associations between clinically important PRO domains and patient outcomes were generally moderate in size, with HR estimates ranging from 1.03 to 1.07. Importantly, this translated to the advanced‐stage patients without any clinically important PRO domains at baseline having a 15% higher overall survival rate at 24 months than the patients with three or more clinically important domains at baseline, underscoring the potential clinical importance of the study findings. Finally, we emphasise that the findings from this study require prospective evaluation and that we strongly support current research aimed at (1) identifying the most essential items from the EORTC QLQ‐C30, as the 30‐item survey may contribute to survey fatigue^54^, ^55^, ^56^, ^57^; (2) exploring the impact of language and cultural factors on patient interpretation of PRO surveys, to ensure generalizability and cross‐cultural validity^58^; and (3) exploring the use of other PRO tools (e.g., the EORTC QLQ‐BR23 or FACT‐B).^59^, ^60^

In conclusion, the findings from this study support the potential value of prospectively evaluating the use of validated clinical thresholds of the EORTC QLQ‐C30 scales in routine breast cancer care to identify actionable insights that could lead to improvement in patients' QoL, survival, and AE outcomes. Notably, over 40% of early‐ and advanced‐stage breast cancer patients reported three or more clinically important PRO domains, with physical function, emotional function, financial difficulty, pain, and dyspnoea among the most frequently reported as clinically important.

## AUTHOR CONTRIBUTIONS

**Bradley D. Menz:** Conceptualization; investigation; writing – original draft; methodology; validation; visualization; writing – review and editing; software; formal analysis; project administration; data curation; resources; funding acquisition. **Natansh D. Modi:** Writing – review and editing; validation; investigation; supervision; resources; methodology. **Ahmad Y. Abuhelwa:** Writing – review and editing; validation; investigation; resources. **Nicole M. Kuderer:** Writing – review and editing; validation; investigation. **Gary H. Lyman:** Writing – review and editing; validation; investigation. **Sandra M. Swain:** Writing – review and editing; validation; investigation. **Ganessan Kichenadasse:** Writing – review and editing; validation; investigation. **Adel Shahnam:** Validation; writing – review and editing; investigation. **Mark Haseloff:** Writing – review and editing; validation; investigation. **Agnes Vitry:** Writing – review and editing; validation; investigation. **Elke Rammant:** Validation; investigation; writing – review and editing. **Imogen Ramsey:** Investigation; validation; writing – review and editing. **Raymond J. Chan:** Investigation; writing – review and editing; validation. **Ross A. McKinnon:** Formal analysis; methodology; investigation; writing – review and editing. **Andrew Rowland:** Supervision; investigation; methodology; writing – review and editing; formal analysis. **Michael J. Sorich:** Supervision; writing – review and editing; methodology; investigation; formal analysis; resources. **Ashley M. Hopkins:** Conceptualization; investigation; funding acquisition; validation; methodology; supervision; writing – review and editing; formal analysis; resources.

## FUNDING INFORMATION

The PhD scholarship of Bradley D. Menz is supported by the National Health and Medical Research Council (APP2030913). Ashley M. Hopkins holds an Emerging Leader Investigator Fellowship from the National Health and Medical Research Council, Australia (APP2008119), and for this research reports support from The Hospital Research Foundation (2023‐S‐DTFA‐005) and Flinders Foundation. Natansh D. Modi's salary is supported by funding from The Hospital Research Foundation (2023‐S‐DTFA‐005) and Tour De Cure (RSP‐117‐FY2023). Micheal J. Sorich is supported by a Beat Cancer Research Fellowship from the Cancer Council South Australia. The funders had no role in considering the study design or in the collection, analysis, interpretation of data, writing of the report, or decision to submit the article for publication.

## CONFLICT OF INTEREST STATEMENT

Andrew Rowland and Micheal J. Sorich are recipients of investigator‐initiated funding for research outside the scope of the current study from AstraZeneca, Boehringer Ingelheim, Pfizer, and Takeda. Ashley M. Hopkins is a recipient of investigator‐initiated funding for research outside the scope of the current study from Boehringer Ingelheim. Andrew Rowland is a recipient of speaker fees from Boehringer Ingelheim and Genentech outside the scope of the current study. Nicole M. Kuderer reports receiving consulting fees from AstraZeneca, Janssen, Pfizer Inc., Bristol Myers Squibb, BeyondSpring Inc., G1 Therapeutics, Inc., Sandoz, Seagen Inc., and Fresenius Kabi outside the submitted work. Gary H. Lyman reported receiving consulting fees from AstraZeneca, Sandoz, G1 Therapeutics, Inc., BeyondSpring Inc., and Fresenius Kabi outside the submitted work. Sandra M. Swain reports a grant to the institution and paid non‐promotional speaking, consulting, and in‐kind manuscript writing monies from Genentech–Roche. Swain also reports grants to the institution from Kailos Genetics and consulting fees paid from Sanofi, AstraZeneca, Daiichi Sankyo, Merck, Molecular Templates, Chugai, Tersera, and Napo Pharmaceuticals. SM. Swain reports a board of director position for Seagen with stock (discontinued 12.23) and Immunome with stock options. Sandra M. Swain reports support for attending meetings and travel from Sanofi, Daiichi Sankyo, and Roche/Genentech. The author team has no other support, financial relationship, or other relationship activities that could appear to have influenced the submitted work.

## Supporting information


**DATA S1.** Supporting Information.

## Data Availability

This publication is based on research using data from Lilly, Pfizer, and Roche that has been made available through Vivli, Inc. Vivli has not contributed to or approved, and is not in any way responsible for the contents of this publication. The data used in this study is available for request with a suitable research proposal at Vivli.org. The source code used to conduct the analyses is available at the following URL: https://github.com/MenzBD/Patient-reported-outcomes-analysis.
