# Supplementary material for: Patient‐reported outcome thresholds and their associations with survival, adverse events, and quality of life in a pooled analysis of breast cancer trials
Source: Int J Cancer. 2025 Jun 21;157(10):2135–45. doi: 10.1002/ijc.70020 (PMC12439079; doi:10.1002/ijc.70020)
Supplement: Supplementary file 1 — DATA S1. Supporting Information. [file IJC-157-2135-s001.pdf]

Supplementary File for Associations with Survival, Adverse Events, and Quality of Life in a Pooled Analysis of Breast Cancer Trials

Bradley D. Menz, Natansh D. Modi, Ahmad Y. Abuhelwa, Nicole M. Kuderer, Gary H. Lyman, Sandra M. Swain, Ganessan Kichenadasse, Adel Shahnam, Mark Haseloff, Agnes Vitry, Elke Rammant, Imogen Ramsey, Raymond J. Chan, Ross A. McKinnon, Andrew Rowland, Michael J. Sorich, Ashley M. Hopkins

**Supplementary** table 1. Thresholds used to define clinically important patient-reported outcome domains	**Page 3**
**Supplementary table 2.** Summary of patient characteristics by disease stage.	**Page 4**
**Supplementary table 3.** Summary of patient characteristics by study in the early-stage cohort	**Page 5**
**Supplementary table 4.** Summary of patient characteristics by study in the advanced-stage cohort	**Page 6 - 7**
**Supplementary table 5.** Patient-reported outcome domain scales presented as median (IQR) by disease stage	**Page 8 - 9**
**Supplementary table 6.** Summary of patient characteristics by the number of clinically important patient reported outcome domains in the early-stage cohort	**Page 10**
**Supplementary table 7.** Summary of patient characteristics by the number of clinically important patient reported outcome domains in the advanced-stage cohort	**Page 11**
**Supplementary figure 1.** Kaplan-Meier estimates of (A) overall survival and (B) invasive-disease-free survival in the early-stage cohort by the number of clinically important patient-reported outcome domains	**Page 12**
**Supplementary table 8.** Kaplan-Meier estimates on the number of clinically important PRO domains with study outcome measures	**Page 13**
**Supplementary figure 2.** Forest plot showing the associations between overall survival and the number of clinically important patient-reported outcome domains by study, in the early-stage cohort.	**Page 14**
**Supplementary figure 3.** Forest plot showing the associations between overall survival and the number of clinically important patient-reported outcome domains by study, in the advanced-stage cohort.	**Page 14**
**Supplementary figure 4.** Forest plot showing the associations between invasive- disease-free survival and the number of clinically important patient-reported outcome domains by study, in the early-stage cohort.	**Page 15**
**Supplementary figure 5.** Forest plot showing the associations between progression-free survival and the number of clinically important patient-reported outcome domains by study, in the advanced-stage cohort.	**Page 15**
**Supplementary figure 6.** Forest plot showing the associations between grade ≥3 adverse events and the number of clinically important patient-reported outcome domains by study, in the early-stage cohort.	**Page 16**
**Supplementary figure 7.** Forest plot showing the associations between grade ≥3 adverse events and the number of clinically important patient-reported outcome domains by study, in the advanced-stage cohort.	**Page 16**
**Supplementary figure 8.** Forest plot showing the associations between serious adverse events and the number of clinically important patient-reported outcome domains by study, in the early-stage cohort.	**Page 17**

**Supplementary figure 9.** Forest plot showing the associations between serious adverse events and the number of clinically important patient-reported outcome domains by study, in the advanced-stage cohort.	**Page 17**
**Supplementary table 9.** Associations between each of the individual clinically important patient-reported outcome domains and overall survival	**Page 18**
**Supplementary table 10.** Associations between each of the individual clinically important patient-reported outcome domains and	**Page 19**
**Supplementary table 11.** Associations between each of the individual clinically important patient-reported outcome domains and grade ≥3 adverse events	**Page 20**
**Supplementary table 12.** Associations between each of the individual clinically important patient-reported outcome domains and serious adverse events	**Page 21**
**Supplementary figure 10.** Calibration curve of the random forest quality of life prediction model comparing the mean predicted quality of life scores generated by the model with the average quality of life scores reported by	**Page 22**

Supplementary table 1. Thresholds used to define clinically important patient-reported outcome domains

EORTC QLQ-C30 domain	Clinically important threshold
Functional domains	
Physical Function	83
Role Functioning	58
Emotional Functioning	58
Cognitive Function	71
Social function	75
Symptomatic domains	
Fatigue	39
Pain at Baseline	25
Nausea and vomiting	8
Insomnia	50
Dyspnoea	17
Appetite loss	50
Constipation	50
Diarrhoea	17
Financial Difficulty	17

Supplementary table 2. Summary of patient characteristics by disease stage.

	Early-stage cohort (n = 5,893)	Advanced-stage cohort (n = 2,651)	P-value
**Study**			< 0.001
APHINITY	4,432 (75%)	0 (0%)	
KATHERINE	1,461 (25%)	0 (0%)	
MONARCH1	0 (0%)	132 (5%)	
MONARCH2	0 (0%)	669 (25%)	
MONARCH3	0 (0%)	493 (19%)	
NEXTMONARCH1	0 (0%)	234 (9%)	
PALOMA3	0 (0%)	521 (20%)	
TH3RESA	0 (0%)	602 (23%)	
**Age**	51 (43 - 59)	58 (50 - 66)	< 0.001
**Sex**			0.41
Female	5,877 (100%)	2,647 (100%)	
Male	16 (<1%)	4 (<1%)	
**Race**			< 0.001
White	4,482 (76%)	1,832 (69%)	
Asian	919 (16%)	574 (22%)	
Other	337 (6%)	137 (5%)	
Unknown	155 (3%)	108 (4%)	
**Estrogen receptor status**			< 0.001
Positive	3,899 (66%)	2,314 (87%)	
Negative	1,992 (34%)	309 (12%)	
Missing	2 (<1%)	28 (1%)	
**Progesterone receptor status**			< 0.001
Positive	2,963 (50%)	1,749 (66%)	
Negative	2,907 (49%)	825 (31%)	
Missing	23 (<1%)	77 (3%)	
**HER2 status**			< 0.001
Positive	5,893 (100%)	602 (23%)	
Negative	0 (0%)	2,049 (77%)	
**ECOG-PS**			< 0.001
0	5,177 (88%)	1,480 (56%)	
≥1	673 (11%)	1,164 (44%)	
Missing	43 (1%)	7 (<1%)	
**Body mass index**			0.027
Median (IQR)	25 (22 - 29)	25 (22 - 30)	
Missing	32 (1%)	58 (2%)	
**Comorbidity count**	2 (1 - 4)	4 (2 - 8)	< 0.001

ECOG-PS: Eastern Cooperative Oncology Group performance status; HER2 Human epidermal growth factor receptor 2; IQR: interquartile range

Supplementary table 3. Summary of patient characteristics by study in the early-stage cohort

	Total (n = 5,893)	APHINITY (n = 4,432)	KATHERINE (n = 1,461)	P-value
**Study**				< 0.001
APHINITY	4,432 (75%)	4,432 (100%)	0 (0%)	
KATHERINE	1,461 (25%)	0 (0%)	1,461 (100%)	
**Age**	51 (43 - 59)	52 (44 - 60)	49 (41 - 57)	< 0.001
**Sex**				0.76
Female	5,877 (100%)	4,421 (100%)	1,456 (100%)	
Male	16 (<1%)	11 (<1%)	5 (<1%)	
**Race**				< 0.001
White	4,482 (76%)	3,399 (77%)	1,083 (74%)	
Asian	919 (16%)	816 (18%)	103 (7%)	
Other	337 (6%)	208 (5%)	129 (9%)	
Unknown	155 (3%)	9 (<1%)	146 (10%)	
**Estrogen receptor status**				< 0.001
Positive	3,899 (66%)	2,868 (65%)	1,031 (71%)	
Negative	1,992 (34%)	1,562 (35%)	430 (29%)	
Missing	2 (<1%)	2 (<1%)	0 (0%)	
**Progesterone receptor status**				< 0.001
Positive	2,963 (50%)	2,146 (48%)	817 (56%)	
Negative	2,907 (49%)	2,285 (52%)	622 (43%)	
Missing	23 (<1%)	1 (<1%)	22 (2%)	
**HER2 status**				
Positive	5,893 (100%)	4,432 (100%)	1,461 (100%)	
**ECOG-PS**				< 0.001
0	5,177 (88%)	3,975 (90%)	1,202 (82%)	
≥1	673 (11%)	414 (9%)	259 (18%)	
Missing	43 (1%)	43 (1%)	0 (0%)	
**Body mass index**				< 0.001
Median (IQR)	25 (22 - 29)	25 (22 - 29)	26 (23 - 30)	
Missing	32 (1%)	16 (<1%)	16 (1%)	
**Comorbidity count**	2 (1 - 4)	2 (1 - 4)	3 (1 - 5)	< 0.001
**Arm of the clinical study**				< 0.001
Trastuzumab	730 (12%)	0 (0%)	730 (50%)	
Trastuzumab + Pertuzumab	2,221 (38%)	2,221 (50%)	0 (0%)	
Trastuzumab + Placebo	2,211 (38%)	2,211 (50%)	0 (0%)	
Trastuzumab emtansine	731 (12%)	0 (0%)	731 (50%)	

ECOG-PS: Eastern Cooperative Oncology Group performance status; HER2 Human epidermal growth factor receptor 2; IQR: interquartile range

Supplementary table 4. Summary of patient characteristics by study in the advanced-stage cohort

	Total (n = 2,651)	MONARCH1 (n = 132)	MONARCH2 (n = 669)	MONARCH3 (n = 493)	NEXTMONARCH (n = 234)	PALOMA3 (n = 521)	TH3RESA (n = 602)	P-value
**Study**								< 0.001
MONARCH1	132 (5%)	132 (100%)	0 (0%)	0 (0%)	0 (0%)	0 (0%)	0 (0%)	
MONARCH2	669 (25%)	0 (0%)	669 (100%)	0 (0%)	0 (0%)	0 (0%)	0 (0%)	
MONARCH3	493 (19%)	0 (0%)	0 (0%)	493 (100%)	0 (0%)	0 (0%)	0 (0%)	
NEXTMONARCH1	234 (9%)	0 (0%)	0 (0%)	0 (0%)	234 (100%)	0 (0%)	0 (0%)	
PALOMA3	521 (20%)	0 (0%)	0 (0%)	0 (0%)	0 (0%)	521 (100%)	0 (0%)	
TH3RESA	602 (23%)	0 (0%)	0 (0%)	0 (0%)	0 (0%)	0 (0%)	602 (100%)	
**Age**	58 (50 - 66)	58 (53 - 67)	60 (51 - 68)	63 (56 - 70)	55 (47 - 65)	57 (49 - 64)	53 (46 - 61)	< 0.001
**Sex**								0.018
Female	2,647 (100%)	132 (100%)	669 (100%)	493 (100%)	234 (100%)	521 (100%)	598 (99%)	
Male	4 (<1%)	0 (0%)	0 (0%)	0 (0%)	0 (0%)	0 (0%)	4 (1%)	
**Race**								< 0.001
White	1,832 (69%)	112 (85%)	373 (56%)	288 (58%)	187 (80%)	385 (74%)	487 (81%)	
Asian	574 (22%)	2 (2%)	214 (32%)	148 (30%)	24 (10%)	105 (20%)	81 (13%)	
Other	137 (5%)	6 (5%)	42 (6%)	18 (4%)	17 (7%)	20 (4%)	34 (6%)	
Unknown	108 (4%)	12 (9%)	40 (6%)	39 (8%)	6 (3%)	11 (2%)	0 (0%)	
**Estrogen receptor status**								< 0.001
Positive	2,314 (87%)	131 (99%)	658 (98%)	490 (99%)	232 (99%)	510 (98%)	293 (49%)	
Negative	309 (12%)	1 (1%)	8 (1%)	2 (<1%)	2 (1%)	3 (1%)	293 (49%)	
Missing	28 (1%)	0 (0%)	3 (<1%)	1 (<1%)	0 (0%)	8 (2%)	16 (3%)	
**Progesterone receptor status**								< 0.001
Positive	1,749 (66%)	95 (72%)	510 (76%)	382 (77%)	181 (77%)	361 (69%)	220 (37%)	
Negative	825 (31%)	35 (27%)	140 (21%)	106 (22%)	51 (22%)	142 (27%)	351 (58%)	
Missing	77 (3%)	2 (2%)	19 (3%)	5 (1%)	2 (1%)	18 (3%)	31 (5%)	
**HER2 status**								< 0.001

Positive	602 (23%)	0 (0%)	0 (0%)	0 (0%)	0 (0%)	0 (0%)	602 (100%)	
Negative	2,049 (77%)	132 (100%)	669 (100%)	493 (100%)	234 (100%)	521 (100%)	0 (0%)	
**ECOG-PS**								< 0.001
0	1,480 (56%)	73 (55%)	400 (60%)	296 (60%)	127 (54%)	322 (62%)	262 (44%)	
≥1	1,164 (44%)	59 (45%)	264 (39%)	197 (40%)	107 (46%)	199 (38%)	338 (56%)	
Missing	7 (<1%)	0 (0%)	5 (1%)	0 (0%)	0 (0%)	0 (0%)	2 (<1%)	
**Body mass index**								< 0.001
Median (IQR)	25 (22 - 30)	25 (22 - 29)	25 (22 - 30)	26 (23 - 30)	25 (22 - 29)	26 (23 - 30)	25 (22 - 28)	
Missing	58 (2%)	2 (2%)	9 (1%)	10 (2%)	9 (4%)	3 (1%)	25 (4%)	
**Disease stage: Advanced**	2,651 (100%)	132 (100%)	669 (100%)	493 (100%)	234 (100%)	521 (100%)	602 (100%)	< 0.001
Abemaciclib	79 (3%)	0 (0%)	0 (0%)	0 (0%)	79 (34%)	0 (0%)	0 (0%)	
Abemaciclib-150mg + Fulvestrant-500mg	325 (12%)	0 (0%)	325 (49%)	0 (0%)	0 (0%)	0 (0%)	0 (0%)	
Abemaciclib-150mg + NSAI	328 (12%)	0 (0%)	0 (0%)	328 (67%)	0 (0%)	0 (0%)	0 (0%)	
Abemaciclib-200mg	132 (5%)	132 (100%)	0 (0%)	0 (0%)	0 (0%)	0 (0%)	0 (0%)	
Abemaciclib-200mg + Fulvestrant-500mg	121 (5%)	0 (0%)	121 (18%)	0 (0%)	0 (0%)	0 (0%)	0 (0%)	
Abemaciclib + Loperamide	77 (3%)	0 (0%)	0 (0%)	0 (0%)	77 (33%)	0 (0%)	0 (0%)	
Abemaciclib + Tamoxifen	78 (3%)	0 (0%)	0 (0%)	0 (0%)	78 (33%)	0 (0%)	0 (0%)	
NSAI	165 (6%)	0 (0%)	0 (0%)	165 (33%)	0 (0%)	0 (0%)	0 (0%)	
Palbociclib + Fulvestrant	347 (13%)	0 (0%)	0 (0%)	0 (0%)	0 (0%)	347 (67%)	0 (0%)	
Physicians Choice	198 (7%)	0 (0%)	0 (0%)	0 (0%)	0 (0%)	0 (0%)	198 (33%)	
Placebo + Fulvestrant	174 (7%)	0 (0%)	0 (0%)	0 (0%)	0 (0%)	174 (33%)	0 (0%)	
Placebo + Fulvestrant-500mg	223 (8%)	0 (0%)	223 (33%)	0 (0%)	0 (0%)	0 (0%)	0 (0%)	
Trastuzumab emtansine	404 (15%)	0 (0%)	0 (0%)	0 (0%)	0 (0%)	0 (0%)	404 (67%)	

ECOG-PS: Eastern Cooperative Oncology Group performance status; HER2 Human epidermal growth factor receptor 2; IQR: interquartile range

Supplementary table 5. Patient-reported outcome domain scales presented as median (IQR) by disease stage

	Early-stage cohort (n = 5,893)	Advanced-stage cohort (n = 2,651)	P-value
**Global Health Status at Baseline**			< 0.001
Median (IQR)	75 (58 - 83)	67 (50 - 83)	
Missing	350 (6%)	209 (8%)	
**Physical Function at Baseline**			< 0.001
Median (IQR)	93 (80 - 100)	80 (67 - 93)	
Missing	338 (6%)	208 (8%)	
**Role Functioning at Baseline**			< 0.001
Median (IQR)	83 (67 - 100)	83 (67 - 100)	
Missing	347 (6%)	209 (8%)	
**Emotional Functioning at Baseline**			0.81
Median (IQR)	78 (67 - 89)	75 (58 - 92)	
Missing	346 (6%)	210 (8%)	
**Cognitive Function at Baseline**			< 0.001
Median (IQR)	100 (83 - 100)	83 (67 - 100)	
Missing	343 (6%)	211 (8%)	
**Social function at Baseline**			< 0.001
Median (IQR)	83 (67 - 100)	83 (67 - 100)	
Missing	350 (6%)	213 (8%)	
**Fatigue at Baseline**			< 0.001
Median (IQR)	22 (11 - 33)	33 (22 - 44)	
Missing	342 (6%)	211 (8%)	
**Nausea/vomiting at Baseline**			< 0.001
Median (IQR)	0 (0 - 0)	0 (0 - 17)	
Missing	338 (6%)	208 (8%)	
**Pain at Baseline**			< 0.001
Median (IQR)	17 (0 - 33)	33 (0 - 50)	
Missing	339 (6%)	208 (8%)	
**Dyspnoea at Baseline**			< 0.001
Median (IQR)	0 (0 - 0)	0 (0 - 33)	
Missing	351 (6%)	213 (8%)	
**Insomnia at Baseline**			0.25
Median (IQR)	33 (0 - 33)	33 (0 - 33)	
Missing	347 (6%)	208 (8%)	

**Appetite loss at Baseline**			< 0.001
Median (IQR)	0 (0 - 0)	0 (0 - 33)	
Missing	343 (6%)	209 (8%)	
**Constipation at Baseline**			< 0.001
Median (IQR)	0 (0 - 0)	0 (0 - 33)	
Missing	344 (6%)	212 (8%)	
**Diarrhoea at Baseline**			0.001
Median (IQR)	0 (0 - 0)	0 (0 - 0)	
Missing	350 (6%)	213 (8%)	
**Financial Difficulty at Baseline**			0.006
Median (IQR)	0 (0 - 33)	0 (0 - 33)	
Missing	395 (7%)	216 (8%)	
IQR: interquartile range			

Supplementary table 6. Summary of patient characteristics by the number of clinically important patient reported outcome domains in the early-stage cohort

	**Clinically important domains 0**	**Clinically important domains 1-2**	**Clinically important domains 3+**	**P-value**
	**n=1,093**	**n=1,934**	**n=2,428**	
**Age**	51 (43 - 60)	51 (43 - 59)	51 (43 - 58)	0.6
**Sex**				0.7
Female	1,089 (100%)	1,930 (100%)	2,422 (100%)	
Male	4 (<1%)	4 (<1%)	6 (<1%)	
**Race**				0.076
White	843 (77%)	1,454 (75%)	1,808 (74%)	
Asian	183 (17%)	313 (16%)	406 (17%)	
Other	44 (4%)	125 (6%)	144 (6%)	
Unknown	23 (2%)	42 (2%)	70 (3%)	
**Estrogen receptor status**				0.55
Positive	705 (65%)	1,283 (66%)	1,603 (66%)	
Negative	388 (35%)	649 (34%)	825 (34%)	
Missing	0 (0%)	2 (<1%)	0 (0%)	
**Progesterone receptor status**				0.48
Positive	550 (50%)	946 (49%)	1,232 (51%)	
Negative	541 (49%)	980 (51%)	1,186 (49%)	
Missing	2 (<1%)	8 (<1%)	10 (<1%)	
**HER2 status**				
Positive	1,093 (100%)	1,934 (100%)	2,428 (100%)	
**ECOG-PS**				< 0.001
0	1,032 (94%)	1,729 (89%)	2,039 (84%)	
≥1	56 (5%)	192 (10%)	375 (15%)	
Missing	5 (<1%)	13 (1%)	14 (1%)	
**Body mass index**				< 0.001
Median (IQR)	24 (22 - 28)	25 (22 - 29)	25 (22 - 30)	
Missing	8 (1%)	6 (<1%)	12 (<1%)	
**Comorbidity count**	2 (0 - 3)	2 (1 - 4)	2 (1 - 5)	< 0.001

ECOG-PS: Eastern Cooperative Oncology Group performance status; HER2 Human epidermal growth factor receptor 2; IQR: interquartile range

Supplementary table 7. Summary of patient characteristics by the number of clinically important patient reported outcome domains in the advanced-stage cohort

	Clinically important domains 0	Clinically important domains 1-2	Clinically important domains 3+	P-value
	n=300	n=632	n=1,486	
**Age**	59 (51 - 66)	58 (50 - 66)	58 (50 - 67)	0.62
**Sex**				0.7
Female	300 (100%)	631 (100%)	1,485 (100%)	
Male	0 (0%)	1 (<1%)	1 (<1%)	
**Race**				0.003
White	189 (63%)	397 (63%)	1,051 (71%)	
Asian	83 (28%)	172 (27%)	296 (20%)	
Other	15 (5%)	33 (5%)	81 (5%)	
Unknown	13 (4%)	30 (5%)	58 (4%)	
**Estrogen receptor status**				0.099
Positive	280 (93%)	569 (90%)	1,328 (89%)	
Negative	18 (6%)	56 (9%)	147 (10%)	
Missing	2 (1%)	7 (1%)	11 (1%)	
**Progesterone receptor status**				0.01
Positive	209 (70%)	457 (72%)	975 (66%)	
Negative	81 (27%)	161 (25%)	469 (32%)	
Missing	10 (3%)	14 (2%)	42 (3%)	
**HER2 status**				0.002
Positive	40 (13%)	100 (16%)	304 (20%)	
Negative	260 (87%)	532 (84%)	1,182 (80%)	
**ECOG-PS**				< 0.001
0	237 (79%)	452 (72%)	677 (46%)	
≥1	62 (21%)	179 (28%)	807 (54%)	
Missing	1 (<1%)	1 (<1%)	2 (<1%)	
**Body mass index**				< 0.001
Median (IQR)	25 (22 - 28)	25 (22 - 29)	26 (22 - 30)	
Missing	3 (1%)	6 (1%)	33 (2%)	
**Comorbidity count**	3 (2 - 5)	3 (2 - 6)	5 (2 - 9)	< 0.001

ECOG-PS: Eastern Cooperative Oncology Group performance status; HER2 Human epidermal growth factor receptor 2; IQR: interquartile range

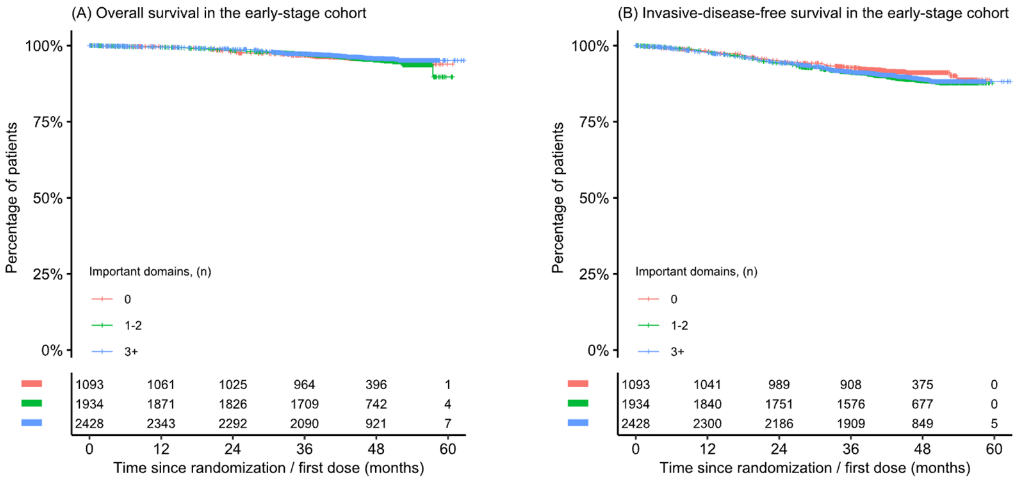

Supplementary figure 1. Kaplan-Meier estimates of (A) overall survival and (B) invasive-disease-free survival in the early-stage cohort by the number of clinically important patient-reported outcome domains

Supplementary table 8. Kaplan-Meier estimates on the number of clinically important PRO domains with study outcome measures

			Early-stage cohort		Advanced-stage cohort	
**Event**	**Important domains, n**	**Time (months)**	**Kaplan-Meier estimates of event (%)**	**% difference**	**Kaplan-Meier estimates of event (%)**	**% difference**
Overall survival	0	24	97.9	-0.9	74.13	14.9
	3+		98.8		59.21	
Invasive-disease-free / progression-free survival*	0	12	98.1	0.3	51.16	9
	3+		97.8		42.18	
Grade ≥3 adverse events	0	6	53.71	3.4	59.05	6.2
	3+		50.3		52.9	
Serious adverse events	0	6	83.76	6.06	92.22	9.7
	3+		77.7%		82.5	

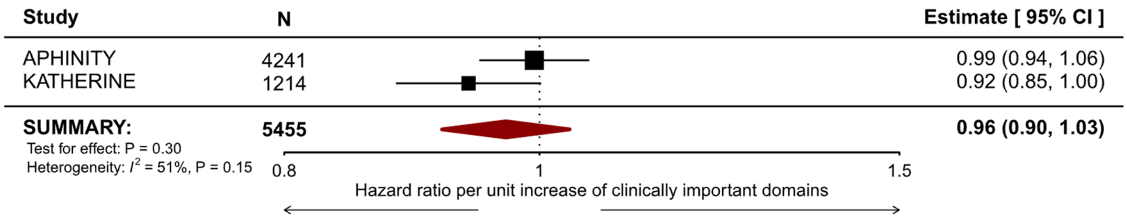

Supplementary figure 2. Forest plot showing the associations between overall survival and the number of clinically important patient-reported outcome domains by study, in the early-stage cohort.

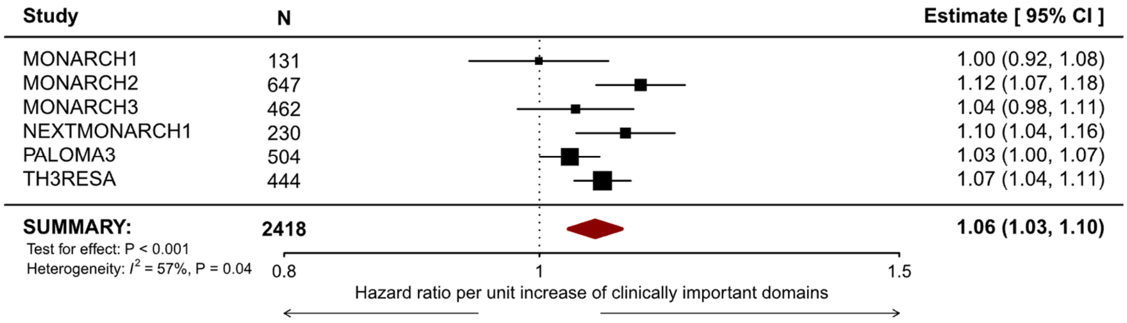

Supplementary figure 3. Forest plot showing the associations between overall survival and the number of clinically important patient-reported outcome domains by study, in the advanced-stage cohort.

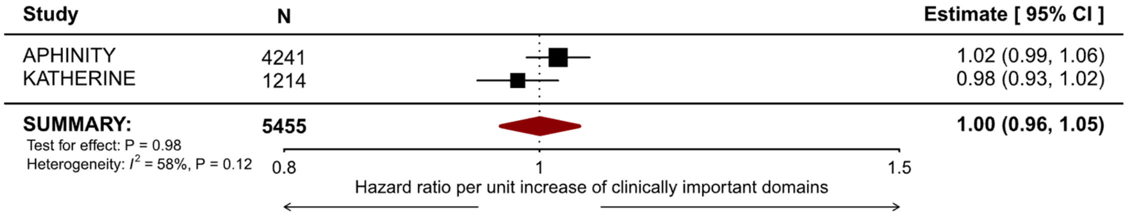

Supplementary figure 4. Forest plot showing the associations between invasive- disease-free survival and the number of clinically important patient-reported outcome domains by study, in the early-stage cohort.

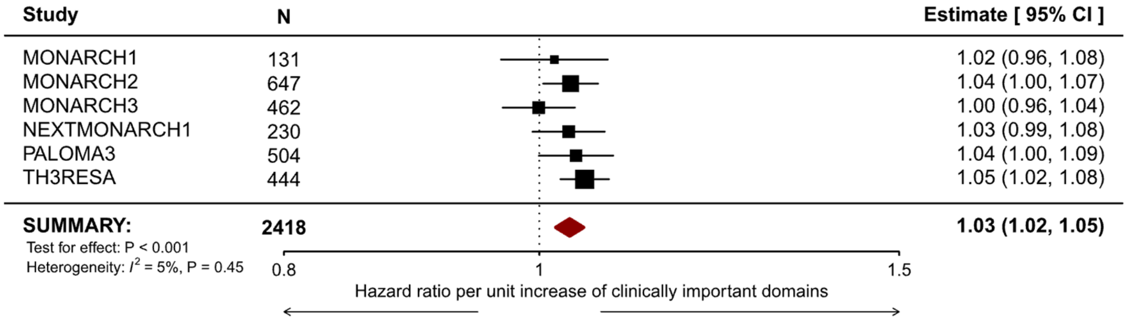

Supplementary figure 5. Forest plot showing the associations between progression-free survival and the number of clinically important patient-reported outcome domains by study, in the advanced-stage cohort.

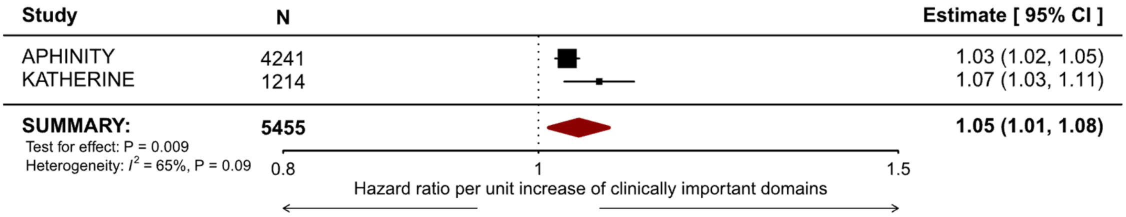

Supplementary figure 6. Forest plot showing the associations between grade ≥3 adverse events and the number of clinically important patient-reported outcome domains by study, in the early-stage cohort.

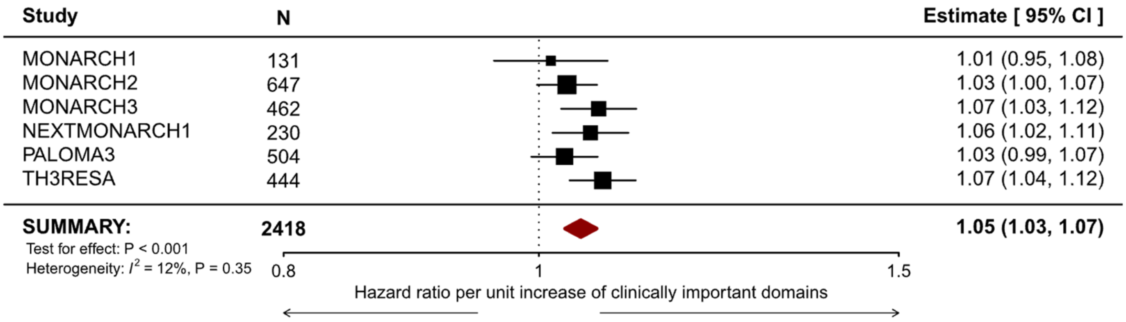

Supplementary figure 7. Forest plot showing the associations between grade ≥3 adverse events and the number of clinically important patient-reported outcome domains by study, in the advanced-stage cohort.

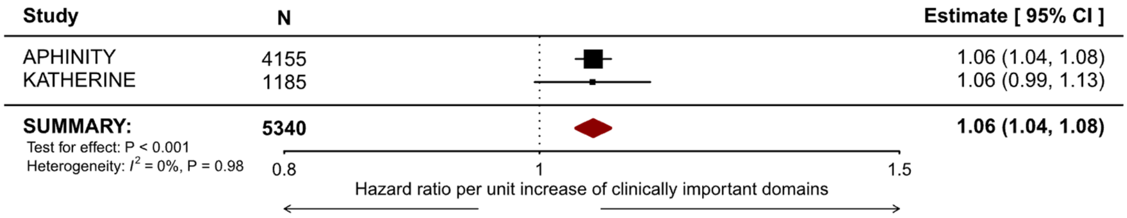

Supplementary figure 8. Forest plot showing the associations between serious adverse events and the number of clinically important patient-reported outcome domains by study, in the early-stage cohort.

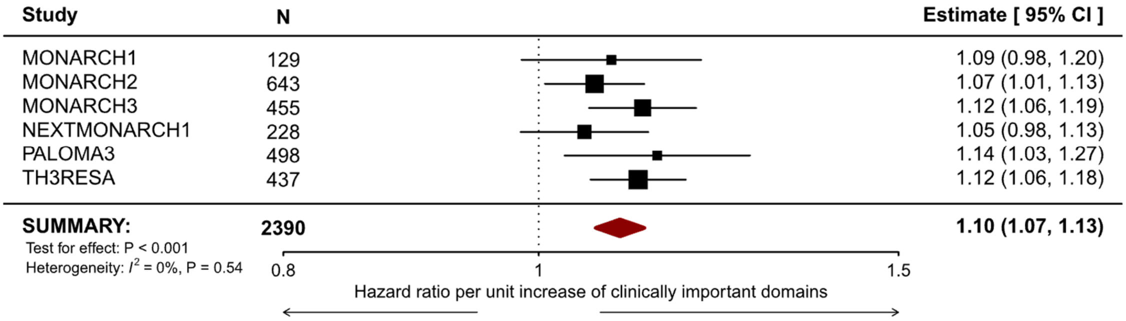

Supplementary figure 9. Forest plot showing the associations between serious adverse events and the number of clinically important patient-reported outcome domains by study, in the advanced-stage cohort.

Supplementary table 9. Associations between each of the individual clinically important patient-reported outcome domains and overall survival

	**Early-stage cohort**		**Advanced-stage cohort**	
**Domain**	**HR (95% CI)**	**P**	**HR (95% CI)**	**P**
Physical function	0.89 (0.66 to 1.22)	0.479	1.41 (1.22 to 1.64)	<0.001
Role function	0.71 (0.47 to 1.07)	0.1	1.26 (1.07 to 1.48)	0.005
Emotional function	0.77 (0.58 to 1.01)	0.06	1.02 (0.88 to 1.17)	0.826
Cognitive function	0.69 (0.48 to 0.99)	0.044	1.03 (0.88 to 1.19)	0.744
Social function	0.93 (0.63 to 1.39)	0.731	1.27 (1.07 to 1.50)	0.006
Fatigue	0.79 (0.54 to 1.16)	0.222	1.34 (1.16 to 1.55)	<0.001
Nausea and vomiting	0.99 (0.66 to 1.46)	0.942	1.29 (1.11 to 1.51)	0.001
Pain	0.75 (0.56 to 1.00)	0.049	1.43 (1.24 to 1.65)	<0.001
Dyspnoea	0.96 (0.70 to 1.33)	0.813	1.29 (1.13 to 1.48)	<0.001
Insomnia	0.97 (0.69 to 1.35)	0.84	0.99 (0.84 to 1.17)	0.904
Appetite loss	1.17 (0.60 to 2.29)	0.652	1.55 (1.28 to 1.88)	<0.001
Constipation	1.2 (0.68 to 2.10)	0.534	1.28 (1.04 to 1.58)	0.02
Diarrhoea	0.92 (0.63 to 1.35)	0.667	0.79 (0.65 to 0.95)	0.012
Financial toxicity	1.32 (1.00 to 1.73)	0.051	1.21 (1.05 to 1.40)	0.007

HR: hazard ratio; 95% CI: 95% confidence interval. Analyses adjusted for age, body mass index, race, Eastern Cooperative Oncology Group - performance status, Human epidermal growth factor receptor 2 status, Estrogen receptor status, Progesterone receptor status, comorbidity count, and line of therapy.
Hazard ratio represents change per unit increase of clinically important PRO domains.

*Supplementary table 10. Associations between each of the individual clinically important patient-reported outcome domains and invasive-disease-free survival or progression free survival**

	**Early-stage cohort**		**Advanced-stage cohort**	
**Domain**	**HR (95% CI)**	**P**	**HR (95% CI)**	**P**
Physical function	1.01 (0.83 to 1.23)	0.934	1.16 (1.04 to 1.31)	0.01
Role function	0.9 (0.71 to 1.14)	0.367	1.12 (0.98 to 1.27)	0.093
Emotional function	0.89 (0.75 to 1.05)	0.174	1.05 (0.94 to 1.17)	0.362
Cognitive function	0.95 (0.77 to 1.17)	0.651	1.01 (0.89 to 1.14)	0.897
Social function	0.96 (0.76 to 1.23)	0.774	1.12 (0.98 to 1.28)	0.109
Fatigue	0.93 (0.74 to 1.16)	0.506	1.22 (1.08 to 1.37)	<0.001
Nausea and vomiting	1.07 (0.84 to 1.36)	0.575	1.2 (1.06 to 1.36)	0.004
Pain	0.94 (0.79 to 1.12)	0.486	1.3 (1.16 to 1.45)	<0.001
Dyspnoea	1.02 (0.83 to 1.25)	0.835	1.06 (0.95 to 1.19)	0.265
Insomnia	0.92 (0.74 to 1.14)	0.441	1.1 (0.97 to 1.25)	0.157
Appetite loss	0.94 (0.59 to 1.51)	0.804	1.18 (1.01 to 1.39)	0.042
Constipation	1.14 (0.78 to 1.65)	0.51	1.28 (1.07 to 1.52)	0.006
Diarrhoea	0.95 (0.75 to 1.21)	0.68	0.91 (0.79 to 1.05)	0.194
Financial toxicity	1.28 (1.08 to 1.53)	0.005	1.03 (0.92 to 1.15)	0.66

HR: hazard ratio; 95% CI: 95% confidence interval. Analyses adjusted for age, body mass index, race, Eastern Cooperative Oncology Group - performance status, Human epidermal growth factor receptor 2 status, Estrogen receptor status, Progesterone receptor status, comorbidity count, and line of therapy.
Hazard ratio represents change per unit increase of clinically important PRO domains.
* Invasive-disease-free survival assessed in the early-stage cohort and progression free survival assessed in the advanced stage cohort.

Supplementary table 11. Associations between each of the individual clinically important patient-reported outcome domains and grade ≥3 adverse events

	Early-stage cohort		Advanced-stage cohort	
**Domain**	**HR (95% CI)**	**P**	**HR (95% CI)**	**P**
Physical function	1.18 (1.08 to 1.29)	<0.001	1.22 (1.07 to 1.38)	0.003
Role function	1.15 (1.05 to 1.27)	0.004	1.37 (1.18 to 1.58)	<0.001
Emotional function	1.08 (1.00 to 1.16)	0.044	1.07 (0.95 to 1.21)	0.245
Cognitive function	1.08 (0.98 to 1.18)	0.128	1.04 (0.91 to 1.18)	0.591
Social function	1.1 (0.99 to 1.22)	0.088	1.28 (1.10 to 1.49)	0.001
Fatigue	1.16 (1.06 to 1.28)	0.002	1.29 (1.14 to 1.46)	<0.001
Nausea and vomiting	1.1 (0.98 to 1.22)	0.093	1.2 (1.05 to 1.37)	0.008
Pain	1.1 (1.01 to 1.18)	0.02	1.3 (1.15 to 1.47)	<0.001
Dyspnoea	1.1 (1.01 to 1.21)	0.035	1.13 (1.00 to 1.27)	0.047
Insomnia	1.16 (1.05 to 1.27)	0.002	1.13 (0.98 to 1.30)	0.101
Appetite loss	1.21 (1.01 to 1.45)	0.038	1.17 (0.98 to 1.40)	0.08
Constipation	1.25 (1.06 to 1.47)	0.008	1.15 (0.95 to 1.40)	0.158
Diarrhoea	1.09 (0.98 to 1.21)	0.109	1.06 (0.92 to 1.23)	0.406
Financial toxicity	0.99 (0.91 to 1.06)	0.709	0.93 (0.82 to 1.05)	0.228

HR: hazard ratio; 95% CI: 95% confidence interval. Analyses adjusted for age, body mass index, race, Eastern Cooperative Oncology Group - performance status, Human epidermal growth factor receptor 2 status, Estrogen receptor status, Progesterone receptor status, comorbidity count, and line of therapy.
Hazard ratio represents change per unit increase of clinically important PRO domains.

Supplementary table 12. Associations between each of the individual clinically important patient-reported outcome domains and serious adverse events

	Early-stage cohort		Advanced-stage cohort	
**Domain**	**HR (95% CI)**	**P**	**HR (95% CI)**	**P**
Physical function	1.3 (1.14 to 1.49)	<0.001	1.59 (1.25 to 2.01)	<0.001
Role function	1.35 (1.17 to 1.56)	<0.001	1.77 (1.40 to 2.23)	<0.001
Emotional function	1.01 (0.90 to 1.13)	0.879	1.09 (0.89 to 1.35)	0.409
Cognitive function	1.14 (0.99 to 1.32)	0.064	1.08 (0.86 to 1.36)	0.513
Social function	1.29 (1.10 to 1.51)	0.002	1.48 (1.16 to 1.89)	0.002
Fatigue	1.37 (1.19 to 1.58)	<0.001	1.51 (1.21 to 1.88)	<0.001
Nausea and vomiting	1.23 (1.04 to 1.44)	0.013	1.45 (1.16 to 1.82)	0.001
Pain	1.2 (1.06 to 1.35)	0.003	1.65 (1.32 to 2.06)	<0.001
Dyspnoea	1.3 (1.13 to 1.49)	<0.001	1.32 (1.07 to 1.63)	0.011
Insomnia	1.26 (1.09 to 1.45)	0.001	1.18 (0.93 to 1.50)	0.172
Appetite loss	1.31 (1.00 to 1.72)	0.048	1.42 (1.07 to 1.87)	0.014
Constipation	1.56 (1.24 to 1.97)	<0.001	1.08 (0.78 to 1.49)	0.658
Diarrhoea	1.08 (0.92 to 1.27)	0.362	0.96 (0.74 to 1.25)	0.767
Financial toxicity	0.99 (0.88 to 1.12)	0.896	1.14 (0.92 to 1.42)	0.227

HR: hazard ratio; 95% CI: 95% confidence interval. Analyses adjusted for age, body mass index, race, Eastern Cooperative Oncology Group - performance status, Human epidermal growth factor receptor 2 status, Estrogen receptor status, Progesterone receptor status, comorbidity count, and line of therapy.
Hazard ratio represents change per unit increase of clinically important PRO domains.

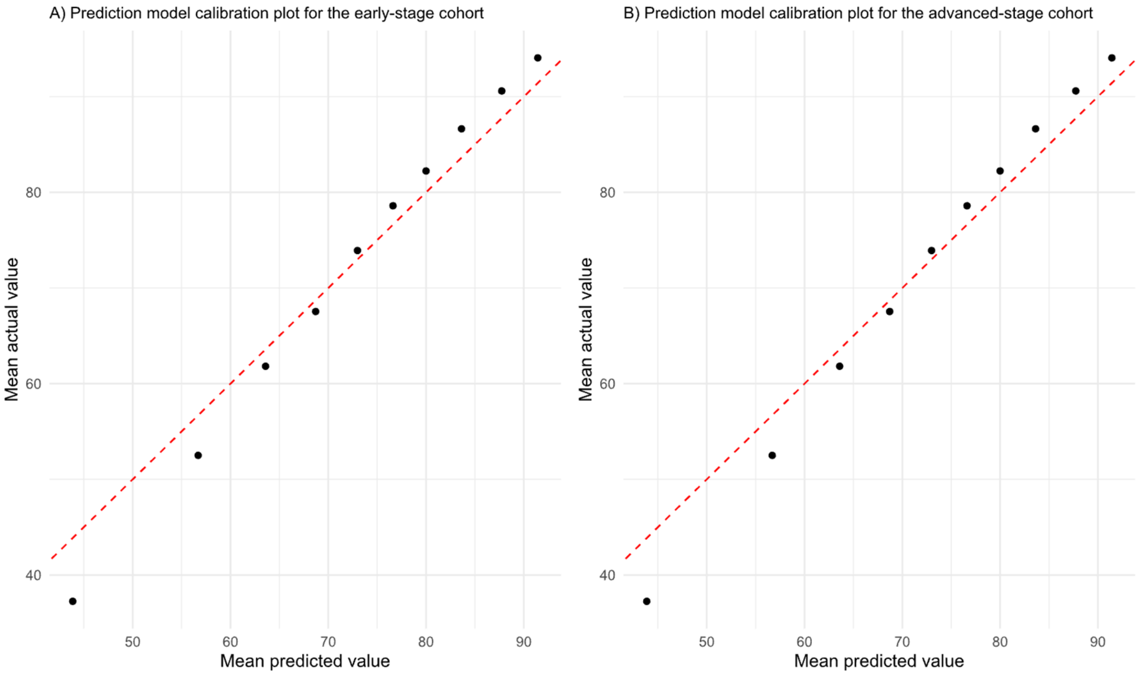

Supplementary figure 10. Calibration curve of the random forest quality of life prediction model comparing the mean predicted quality of life scores generated by the model with the average quality of life scores reported by patients in the (A) early-stage cohort and (B) advanced-stage cohort
